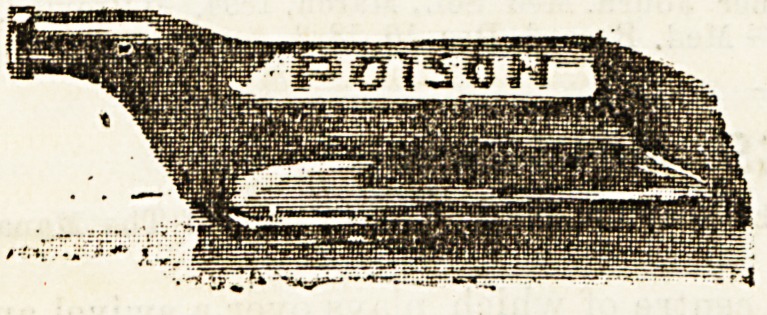# New Appliances and Things Medical

**Published:** 1894-05-19

**Authors:** 


### NEW APPLIANCES AND THINGS MEDICAL.

[All preparations, appliances, novelties, &c., of which a notice is desired, should be sent for the Editor, to care of The Manager, 428, Strand, London, W.C.]

### SELF-ADJUSTING ABDOMINAL BELT.

(H. Wilkinson, 115, Mansfield Road, Nottingham.)

Quite a new form of abdominal belt has been manufactured by the above firm. The great advantage which this belt offers over other designs is the possession of the so-called "Lupus Thigh Band." The object of this band is to prevent shifting or working up, and to afford greater support to the lower part of the abdomen. It is formed of a continuous elastic strand, the centre of which plays over a swivel and pully in front of the belt, and works with every movement of the body. The belt itself is capable of great expansion, and that, too, without the inconvenience of unlacing; and in this respect is very useful during pregnancy. The only disadvantage that we can detect is that the belt is rather cumberous and heavy. It would, however, be difficult to construct a belt of similar supporting powers and of similar strength without the above objection.

### LEMON SYRUP.

(H. W. Carter and Co., Old Refinery, Bristol.)

Messrs. Carter, the well-known manufacturers of non-alcoholic and teetotal beverages, have added one more useful item to their already goodly list. The lemon syrup to which we refer is a highly concentrated, pale yellow-coloured fluid, possessing the aroma and taste of fresh lemons. It will stand a greater degree of dilution than any other similar syrup with which we are acquainted, and when mixed with plain or aerated water constitutes as delicious a lemonade or lemon squash as those which are made from the fresh lemon. In the Old Refinery, Bristol, there is an interesting and specially constructed room for the manufacture of this and similar syrups. The object of the room is to ensure absolute cleanliness in all the stages of the manufacture. Thus the room is so constructed that there are no angles for the accumulation of dirt. The floor, walls, and ceiling are of cement, and the door and fittings of iron, while the utensils for the reception of the syrups are bell-shaped pans of enamelled iron. By means of a hydrant and a full pressure of water it is possible to flush the whole place end to end, and thus ensure as absolute a degree of cleanliness as is desirable in the preparation of this and similar syrups.

### DISINFECTANTS.

(The Nobes Disinfecting Fluid and Powder Company, Willesden Junction, N.W.)

The above firm have forwarded us samples of three of their disinfecting media, namely: (1) The Primrose Eucalyptus Fluid; (2) Violet Antiseptic Fluid; (3) The Zymotic Eucalyptus Powder. The first-named of these preparations is an amber-coloured fluid, having rather a pleasant odour of the purer forms of eucalyptus, and free from the disagreeable odour which attaches to the commercial and crude samples of this oil. There can be no doubt that it is a powerful oxydising agent, and is eminently suited for cleaning contaminated articles from the sick room. The second preparation on the list is also a powerful oxydising agent, and within limits is non-poisonous; it has considerable advantages as a cleansing and antiseptic lotion for foul wounds and unhealthy sores, whether in hospital or veterinary practice. The eucalyptus powder presents an admirable form of antiseptic, which can be applied in the dry form, and thus avoid the damage which fluid antiseptics must of necessity effect.

### NOVEL POISON BOTTLE.

(R. H. Quine, 1, Leaf Square, Pendleton, Manchester.)

Mr. Quine seems to have designed a poison bottle which is just what a poison bottle should be, namely, absolutely different, and unlike any other bottle which has preceded it. There can be no doubt that if it were made compulsory by law that all poisons should be dispensed in bottles of some distinctive shape, such as that designed by Mr. Quine, within six months an appreciable and notable decrease in the number of deaths from accidental poisoning would be observed in the Registrar-General's returns. The bottle we refer to has the following characteristics. The bottle will only stand in one position, namely, on its side, and in this position the label and the embossed word "Poison" can be distinctly read. The peculiarity of the neck, which is elevated to a short height above the rest of the bottle, makes it impossible to mistake, it for an ordinary medicine bottle lying on its side and this arrangement of the neck makes the bottle exceedingly useful for liniments and paints which have to be applied by the brush, since even when the bottle is nearly empty it is possible to reach the fluid with even a very short brush. At present the bottles are manufactured either in blue or white, but the colour is probably a matter of no great importance, but the designer suggests that prescriptions and strong poisons should be dispensed in the blue variety, and simple household remedies, such as soap liniment, camphorated oil, &c., in plain, clear glass. One great advantage for the poorer classes that this variety has over the ordinary blue corrugated poison bottle is that it is no more expensive than the ordinary medicine bottle. This new bottle is so clever and useful an invention that we wish the designer every success in his venture.

### AROMATIC OXIDATES.

(Household Disinfectant Company, 10, Kensington High Street, W.)

The above firm have forwarded for our inspection a little contrivance which they assert creates peroxide of hydrogen on being hung to a chandelier or elsewhere, where it can freely vibrate in the air. It is composed of a white porcellaneous substance, said to be powerfully charged with purely non-poisonous vegetable oils, having the power to create peroxide of hydrogen; and moulded in the form of a bunch of grapes, the stalk of which is twisted wire, and by means of which it may be suspended. The antiseptic properties of vegetable oils, such as eucalyptus, cloves, &c., are well known; but whether an amount of peroxide of hydrogen emanates from this preparation sufficient to be of real service is somewhat problematical, and its presence might give a false sense of security against infection where no security exists. We would personally prefer to run the risk of infection by means of impure air rather than enjoy immunity while breathing air impregnated with the odours of aromatic oxidates.

### FLUID EXTRACT OF COCILLANA.

(Park, Davis, and Co., 43 and 44, Holborn Viaduct, E.C.)

This is a new remedy introduced from Bolivia, where it has long been employed by the natives as an emetic of high repute. Dr. Rusby, during his explorations in South America, was made acquainted with this drug, and was enabled to locate the bark, from which it is prepared, as belonging to a species of the genus Guarea of the natural order of Meliaceæ. The exact species appears still to be undetermined. The natives are in the habit of chewing the bark when they require the services of an emetic, and the action is reliable and constant. Since the drug has been submitted to careful clinical investigation its services as an emetic have been dispensed with, and it has been brought into requisition in cases of pulmonary disorder where expectorants are indicated. Although recommended by some of its exponents as a useful drug in all stages of bronchitis and phthisis, from a careful perusal of the literature on the subject we should feel inclined to recommend it only in those cases of bronchitis which have relapsed into a chronic condition with scanty and thick sputum. Amongst the virtues which are claimed for this preparation may be enumerated a tonic influence on appetite, a checking action on the night sweats of phthisis, and a laxative effect on the bowels.

## Figures and Tables

**Figure f1:**